# Thyroid hormones and neurological impairments during the first two days after stroke: A pilot study

**DOI:** 10.1016/j.heliyon.2025.e41921

**Published:** 2025-01-11

**Authors:** Kobra Akhoundzadeh, Ashraf Zarvani, Fatemeh Mohajerani, Sakineh Shafia

**Affiliations:** a Department of Physiology, Qom University of Medical Sciences, Qom, Iran; b Department of Neurology, Mazandaran University of Medical Sciences, Sari, Iran; c Department of Neurology, Yasuj University of Medical Sciences, Yasuj, Iran; d Immunogenetics Research Center, Department of Physiology, Mazandaran University of Medical Sciences, Sari, Iran

**Keywords:** Thyroid hormone, Neurological impairments, Stroke

## Abstract

Despite plenty of studies on the change in thyroid hormones after stroke, there is still no consensus for a comprehensive conclusion and clinical application. This pilot study aimed to investigate the change in thyroid hormones and its association with neurological impairments during the first two days after the stroke. This was a cross-sectional descriptive and analytical study. Neurological deficits were assessed using the National Institutes of Health Stroke Scale (NIHSS). In addition, blood samples were collected for TSH, T_3,_ and T_4_. P value < 0.05 was considered significant. Fifty patients (42 % men) with a mean age of 69.73 (SD ± 12.31) were included. The majority of patients had a high level of TSH and T_4_, and a normal level of T_3_ on the first and second days of hospitalization. 33.3 and 20.9 percent of patients had NIHSS scores >10 on the first and second day respectively. According to the multivariate analysis, high T_4_ hormone was as a predictor of NIHSS score >10. There was no association between thyroid hormones and NIHSS at the admission. In addition, there was no relationship between thyroid hormones and TSH on the second day. According to the results, stroke affects the levels of thyroid hormones and the pituitary–thyroid axis. Because of the diversity in results of existing studies, further clinical research with the same methodology and data analysis is needed to find consistent results on the association between thyroid hormones and neurological impairments. Monitoring thyroid status in stroke patients may be recommendable.

## Highlights

• The levels of TSH and T_4_ hormones are altered after stroke.
• High T_4_ hormone could be a predictor of moderate to severe strokes.
• The stroke probably affects the negative feedback of the pituitary-thyroid axis.

## 1 Introduction

Stroke remains a world challenge, and therefore more effective strategies for preventing and managing stroke are still required [1]. Although plenty of studies have been done on the change in thyroid hormones after stroke, there is still no consensus for a comprehensive conclusion and clinical application [[2], [3], [4]]. Non-thyroidal illness syndrome (NTIS) and low T_3_ syndrome are some definitions applied for the change in thyroid hormones after illnesses such as stroke [5,6]. NTIS described the abnormality of thyroid hormones, particularly T_3_ following illness without thyroid dysfunction [5]. According to the evidence, T_3_ can be considered as a prognostic biomarker in patients with ischemic stroke [7]. Besides T_3_ and T_4_, TSH is also affected by stroke [3]. It seems that TSH alteration after stroke can be related to central hypothyroidism reported in many illness models. Central hypothyroidism is due to diminished hypothalamic TRH in the brain [5].

In spite of numerous studies evaluating thyroid hormone change after stroke, it is still unknown whether thyroid hormone alternations have beneficial or harmful effects [[2], [3], [4], [5],[8], [9], [10], [11]]. These ambiguities make thyroid hormone replacement therapy controversial in clinics [5]. Also, it is unclear which hormone (T_3_ or T_4_) can be suitable to be used [5,12]. Nevertheless, some studies showed therapeutic effects of T_3_ and T_4_ administration in experimental studies [[13], [14], [15]].

Some of the mechanisms that have been supposed for beneficial effects of thyroid hormones on stroke include anti-edema action through the inhibition of aquaporin-4 water channel expression [16], upregulation of BDNF and GDNF in the hippocampus [17], inhibition of apoptosis, and suppression of inflammatory reactions [14]. Conversely, according to some studies, hypothyroid animals subjected to cerebral ischemia showed the protective effects of hypothyroidism such as reduced metabolic activity, apoptosis, necrosis, oxidative stress, cell death [18,19], and glutamate release [20]. Similarly, both negative and positive associations have been shown between thyroid hormones states and post-stroke neurological impairments in human studies [[2], [3], [4]].

Taken together the results related to thyroid hormone changes after stroke and the association between thyroid hormone levels and neurological impairments in stroke patients are controversial and not yet useful for clinical application. Also, as far as we found the majority of existing studies assessed thyroid hormone changes on the admission day of the stroke while the peak of many stroke-related changes such as inflammation or cerebral edema is observed on day-2 or later [21,22]. This pilot study aimed to investigate the change in thyroid hormones and its association with post-stroke neurological impairments during the first two days after stroke.

## 2 Methods and materials

This was a cross-sectional descriptive and analytical study done in the Department of Neurology of Bouali Hospital in Sari affiliated to Mazandaran University of Medical Sciences, Iran. Patients with acute cerebral ischemic stroke signs and symptoms diagnosed by neurologist using clinical assessment and computed tomography (CT) or magnetic resonance imaging (MRI) findings were enrolled in the study. Inclusion criteria were new ischemic stroke patients between 18 and 80 years old and without a history of thyroid abnormality and neurological disorders. Hemorrhagic stroke, death, severe complications (such as severe brain edema, haemorrhagic transformation, cardiac arrest, respiratory failure, etc.), bleeding, a history of thyroid abnormality, and use of thyroxine therapy or antithyroid medication were considered as exclusion criteria. None of the patients underwent thrombolytic therapy. The patients were selected using consecutive sampling method between December 2020 and September 2021. The study was approved by Iran National Committee for Ethics in Biomedical Research (Approval Code: IR.MAZUMS.REC.1398.1098 and IR.MAZUMS.REC.1398.397). All methods were performed in accordance with the relevant guidelines and regulations. Informed consent was obtained from all subjects and/or their legal guardian(s). The data were collected anonymously. Neurological deficits were assessed by a neurologist using the National Institutes of Health Stroke Scale (NIHSS) on the admission and the second days of hospitalization (https://www.stroke.nih.gov/documents/NIH_Stroke_Scale_508C.pdf). In addition, data on demographic characteristics and the history of hypertension, diabetes, heart disease, atrial fibrillation, stroke, hyperlipidemia, smoking, and alcohol use were collected.

Blood samplings for TSH, total T_3_, and total T_4_ were gathered at admission and day 2. The serums were frozen at −20C until laboratory measurement. The hormones were measured by enzyme-linked immunosorbent assay (ELISA) kit (Pishtazteb Co. Iran). Normal ranges for TSH, total T_4_, and total T_3_ are 1.1–1.8 mlU/L, 6.4–9.0 μg/dl, and 0.9–1.6 ng/ml respectively.

## 3 Statistical analysis

After testing for normality, non-parametric quantitative data were presented as median (IQR) and qualitative data were presented as frequency and percentages. The groups were compared using Wilcoxon test, or chi-square tests. The correlation between non-parametric data was analyzed using Spearman's tests. Binary logistic regression analysis was performed to determine the predictors of moderate to severe neurological deficits. Data were analyzed using SPSS software version 22. P value < 0.05 was considered statistically significant.

## 4 Results

Fifty patients with available laboratory data and neurological impairment documentation were included, 21 men and 29 women with a mean age of 69.73 (SD ± 12.31). Patient Characteristics are listed in Table 1.

**Table 1:** Characteristics of study patients.

Variables	
Age (mean ± SD)	69.7 ± 12.3
Sex	
Female n %	29 (58 %)
Male n %	21 (42 %)
History of	
HTN n %	33 (78.6 %)
Diabetes n %	21 (51.2 %)
Heart disease n %	18 (38.3 %)
Stroke n %	13 (27.7 %)
Hyperlipidemia n %	18 (38.3 %)
Atrial fibrillation n %	4 (8.5 %)
Smoking n %	3 (5.8 %)
Alcohol n %	0

The frequency and percent of the patients with low, normal, or high levels of TSH and thyroid hormones on the first and second days after stroke are summarized in Table 2. In addition, this table shows the frequency of patients with minor to moderate stroke or moderate to severe stroke. It is noteworthy that the results showed that 27 (56.3 %) and 33 (68.8 %) patients had a minor stroke (NIHSS≤8) on the first and second days respectively.

**Table 2:** Frequency and percent of low, normal, and high levels of TSH and thyroid hormones, and stroke severity on the first and second days.

Variable	Admission day n %	Second day n %
**TSH**		
<1.1 mlu/l	11 (22.4 %)	16 (33.3 %)
Normal	14 (28.6 %)	13 (27.1 %)
>1.8 mlu/l	24 (49 %)	19 (39.6 %)
**T**_**3**_		
< 0.9 ng/mL	3 (6.1 %)	4 (8.3 %)
Normal	37 (75.5 %)	35 (72.9 %)
> 1.6 ng/mL	9 (18.4 %)	9 (18.8 %)
**T**_**4**_		
< 6.4 μg/dl	0 (0 %)	1 (2.1 %)
Normal	17 (34.7 %)	18 (37.5 %)
>9 μg/dl	32 (65.3 %)	29 (60.4 %)
**NIHSS**		
minor to moderate stroke (NIHSS ≤10)	32 (66.7 %)	38 (79.2 %)
moderate to severe stroke (NIHSS >10)	16 (33.3 %)	10 (20.8 %)

The Wilcoxon signed-rank test showed no significant differences between T_3_, T_4_, and TSH levels on the first and second days (Table 3). Although there was no statistically significant difference between the first and second day NIHSS scores, the p-value was near the level of significance (0.052) (Table 3).

**Table 3:** Comparison of thyroid hormones, TSH, and NIHSS between the first and second days.

Variable	Admission time Median (IQR)	Second day Median (IQR)	P-value
**TSH**	1.8 (1.1–2.6)	1.4 (0.90–2.6)	0.71
**T**_**3**_	1.5 (1.1–1.6)	1.2(1.1–1.5)	0.44
**T**_**4**_	9.8 (8–10.7)	9.15 (7.95–10.37)	0.51
**NIHSS**	8 (5–11)	5 (4–9)	0.052

Table 4 presents the associations between thyroid hormones, TSH, and NIHSS analyzed using Spearman's test. There was no relationship between NIHSS and thyroid hormones or TSH. The first-day TSH was correlated with thyroid hormones, while there was no relationship between TSH and thyroid hormones on the second day.

**Table 4:** Associations (correlation coefficient r_s_) between thyroid hormones, TSH, and NIHSS score analyzed using Spearman's test.

		First day				Second day			
		TSH	T_3_	T_4_	NIHSS	TSH	T_3_	T_4_	NIHSS
**First day**	**TSH**	–	−0.37∗∗	−0.29∗	NS	0.61∗∗	NS	NS	NS
	**T**_**3**_	−0.37∗∗	–	0.28∗	NS	−0.30∗	0.38∗∗	NS	NS
	**T**_**4**_	−0.29∗	0.28∗	–	NS	NS	0.38∗∗	0.39∗∗	NS
	**NIHSS**	NS	NS	NS	–	NS	NS	NS	NS
**Second day**	**TSH**	0.61∗∗	−0.30∗	NS	NS	–	NS	NS	NS
	**T**_**3**_	NS	0.38∗∗	0.38∗∗	NS	NS	–	0.49∗∗	NS
	**T**_**4**_	–	–	0.39∗∗	NS	NS	0.49∗∗	–	NS
	**NIHSS**	NS	NS	NS	NS	NS	NS	NS	–

NS not significant, ∗p < 0.05; ∗∗p < 0.01.

The result of univariate and multivariate analysis between thyroid hormone and TSH levels and second-day NIHSS>10 is presented in Table 5. According to the results of univariate analysis, high TSH and high T_4_ on the second day had an association with moderate to severe stroke. However, after multivariate analysis, only high T_4_ remained as a predictor of moderate to severe stroke (β = −2.58, p = 0.018).

**Table 5:** Univariate and multivariate analysis between thyroid hormone and TSH levels and second-day NIHSS>10. The factors that were found to contribute to the NIHSS in the univariate analysis at P values < 0.2 were included in the multivariate. For each variable, the odds ratio (OR), and 95 % confidence interval (CI) are given.

Variable	Univariate analysis			Multivariate analysis		
	Odd ratio (OR)	95 % CI	P value	Odd ratio (OR)	95 % CI	P value
**Age**	1.03	0.94–1.09	0.401	–	–	–
**Sex**	2.88	0.69–12.07	0.147	3.43	0.39–29.95	0.26
**HTN**	1.38	0.275–6.92	0.69	–	–	–
**Diabetes**	1.67	0.34–8.17	0.53	–		–
**Heart disease**	0.24	0.51–1.13	0.071	0.17	0.022–1.26	0.08
**Stroke**	0.40	0.09–1.83	0.29	–	–	–
**Hyperlipidemia**	0.94	0.07–11.97	0.96	–	–	–
**Atrial fibrillation**	0.71	0.06–7.70	0.77	–	–	–
**First day TSH>1.8**	1.11	0.28–4.48	0.88	–	–	–
**Second day TSH>1.8**	5.06	1.11–23.01	0.036	5.01	0.55–45.54	0.15
**First day T**_**3**_**>1.6**	3.75	0.87–16.22	0.077	2.07	0.23–18.74	0.52
**Second day T**_**3**_**>1.6**	1.24	0.22–6.9	0.81	–	–	–
**First day T**_**4**_**>9**	0.69	0.16–2.92	0.62	–	–	–
**Second day T**_**4**_**>9**	5.06	1.11–23.01	0.036	13.22	1.56–111.79	0.018

## 5 Discussion

Our main findings were that the majority of patients had high TSH and T_4_ levels and a normal T_3_ on the admission and second days. High T_4_ hormone on the second day was a predictor of moderate to severe strokes (NIHSS >10). There were no negative expected relationships between TSH and thyroid hormones on the second day.

Although existing studies reported low T_3_ syndrome after illnesses [5,23], our study did not show a low T_3_ level on the admission day except in 3 patients (6 %), and the median T_3_ level remained in the normal range (Table 3). This discrepancy in results may be because of differences in the severity of illness. In our study the majority of patients were admitted with a minor to moderate stroke (NIHSS score ≤10), and severe stroke (NIHSS>20) was just revealed in 3.8 % of patients (Data was not shown). Accordingly, our finding is in agreement with the literature that reported that the degree of serum total T_3_ falls in illness is often proportional to the severity of illness [5]. It is stated that in serious illness, low T_3_ is a compensatory mechanism to save energy and protein [24]. Partly similar to our result, in Chen and colleagues' study mean serum T_3_ concentration after stroke remained in the normal range [23]. Also, Zhang and colleagues showed that the majority of patients with mild stroke (NIHSS <8) had normal T_3_ [25].

Table 4, Table 5 present that there was no association between T_3_ and NIHSS. Although the association between low T_3_ and stroke severity or neurological deficit following stroke was reported in several studies [4,[24], [25], [26], [27], [28]], and some studies showed an inverse association between low T_3_ and post-stroke outcome [29], due to the small number of patients with low T_3_ levels, we were unable to statistically assess the association between low T_3_ levels and stroke severity.

As shown in Table 3, the median TSH level, especially on the first day, tended to the upper limit, and the majority of patients had a high level of TSH. It is reported that TSH level is suppressed after a moderate to severe illness [5], therefore, since in the present study the majority of patients had a minor stroke, TSH suppression was not marked. It is worth mentioning that although, rank Spearman correlation and multivariate analysis did not show a statistically significant relationship between TSH and NIHSS on the second day, based on univariate analysis a high level of TSH was a negative predictor of moderate to severe neurological impairment on the second day (β = −1.62). O’ Keefe and colleagues' study reported no association between NIHSS and TSH [30], however, in Akhound and colleagues' study, patients with higher levels of serum TSH tended to have a milder stroke [31,32]. Also, a meta-analysis study showed elevated TSH may correspond to better functional outcomes [3].

The results of the present study revealed that the T_4_ level, especially on the first day, was higher than normal in over 60 % of patients. Partly in harmony with our finding, it is reported that dependent on the methodology, FT_4_ may show an increase in an illness of a moderate severity [5]. According to some studies, high T_4_ levels and low levels of T_3_ after stroke may be explained by decreased peripheral conversion of T4 to T3. In addition, the reduction in peripheral conversion is related to disease severity [33].

As shown in Table 5, a high level of T_4_ on day 2 was a predictor of moderate to severe neurological deficit. Since the beta coefficient was negative in direction, it indicates that high T_4_ was a negative predictor of neurological deficit. After adjustment, none of the other hormones, including T_3_, T_4_, and TSH on the first day, and T_3_ and TSH on the second day were the predictor of moderate to severe neurological deficit on day 2. It is mentionable that there was no association between moderate to severe neurological deficit and high level of T_4_ on the admission day (data was not shown). The results of the relationship between T_4_ and neurological deficit and stroke outcomes are confusing. Many studies showed no significant association between T_4_ and neurological deficit [9,11,24,25,[34], [35], [36], [37], [38]] and some revealed that lower T_4_ was associated with worse neurological outcomes [39]. However, a meta-analysis reported that patients with poor outcomes had significantly higher T_4_ levels and lower levels of T_3_ [2]. Although the exact mechanisms underlying the adverse effects of thyroid hormones on stroke outcomes are not still understood well, a probable mechanism is the excitement of sympathetic nervous system induced by excessive thyroid hormones. The overactivation of the sympathetic nervous system can lead to a severe hypermetabolic response followed by higher production of Reactive Oxygen Species (ROS) and free radicals and cytotoxicity (see the review article by Murolo et al.)[40].

It seems that the form of thyroid hormone assessed as free or total hormone may be involved in the variability of the results because it is asserted that the change of free thyroid hormones in illness is more modest than that of total thyroid hormone [5]. In the present study, total T_4_ was measured owing to more detectable changes, however, the majority of the mentioned studies measured free T_4_ [9,11,29,[35], [36], [37], [38]]. It is important to mention that in our study, confidence intervals in regression analysis were too wide (Table 5), which means a greater uncertainty level. Since the present study was a pilot study and the sample size was small, it influenced confidence intervals.

According to the results in Table 3, although not statistically significant, all parameters including stroke severity score (NIHSS), T_3_, T_4_, and TSH were coordinately higher on the first day than that on the second day. The reduction in T_3_, T_4_, and TSH from the hyper-acute (within 24 h) to acute (3–5 days) stages of stroke was also reported in Sidorov and colleague's study [26]. Due to the lack of statistically significant results, we cannot draw a conclusion, however, the question arises whether thyroid hormone alterations are affected by stroke severity, or stroke severity is impressed by thyroid hormone alternations.

Another main finding of the present study was that the physiological relationships between TSH and thyroid hormones were preserved on the first day; however, these relationships were not maintained on the second day (Table 4). In other words, negative feedback of pituitary–thyroid axis was not observed on the second day after stroke. Since it is asserted that illnesses could profoundly impress neuroendocrine systems such as the hypothalamic–pituitary–thyroid (HPT) axis [5], disturbance of the relationship between TSH and thyroid hormones may partly be explained. However, it is not clear whether hypothalamic–pituitary–thyroid axis changes after illnesses are a compensating or detrimental response [5].

The studies of the relationship between thyroid hormones and stroke severity and neurological impairment showed different findings. Some factors that may contribute the diverse findings include different assessment scales for neurological impairment (NIHSS, mRs, Scandinavian Stroke Scale, etc.), different categorizations of neurological impairment (minor, mind, moderate, and severe), the form of thyroid hormone (total, free), the time of assessment, and small sample size. Whether correcting thyroid profile with hormone supplementation or antagonism may lead to improved outcomes will require large, prospective, interventional studies. Overall, the relationship between thyroid hormones and strokes seems to be complicated and requires further well-designed investigations.

Lastly, it needs to be mentioned that the data on self-reported smoking and alcohol use (Table 1) seems to be not valid due to patients' reluctant to report, as was also shown in other studies [41,42].

## 6 Limitations and recommendations

Further studies with a well-designed method and increased number of patients should be done to achieve more reliable results. In addition, assessment of hormone levels and neurological impairments over multiple time points is recommended to find their association over time. The lack of neurological outcome assessments at discharge or long time assessments was a limitation of this study.

## 7 Conclusion

According to the results of the present study, stroke affects the TSH and T4 levels, and the negative feedback of the pituitary–thyroid axis. Also, T_4_ can be suggested as a predictor of moderate to severe stroke. However, the T_3_ level was maintained in the normal range and the low T_3_ syndrome was not shown in most patients. These findings support some previous studies and seemingly differ from others. Many confounding factors and study limitations, such as stroke severity, number of patients, thyroid hormone form (free or total), time of assessment, data analysis methods and neurological impairment scales, may have contributed to the difference in the results of these studies. Therefore, further larger and longitudinal studies with precise methodology and proper data analysis techniques are needed to find consistent results on the association between thyroid hormones and neurological impairments and to confirm our findings. Regarding the changes in thyroid hormones following stroke and the possible association between thyroid hormones and neurological deficits, monitoring thyroid status in stroke patients during consecutive days may be recommendable.

## CRediT authorship contribution statement

**Kobra Akhoundzadeh:** Writing – original draft, Conceptualization. **Ashraf Zarvani:** Investigation. **Fatemeh Mohajerani:** Methodology. **Sakineh Shafia:** Supervision.

## Ethics approval and consent to participate

This work approved by Iran National Committee for Ethics in Biomedical Research (record no. IR.MAZUMS.REC.1398.1098, IR.MAZUMS.REC.1398.39, IR.MAZUMS.REC.1397.3291, IR.MAZUMS.REC.1398.6078) was supported by 10.13039/501100004160Mazandaran University of Medical Sciences, Sari, Iran. Informed consent was obtained from all subjects and/or their legal guardian(s)

## Consent for publication

Not applicable.

## Availability of data and materials’ statement

The datasets used and analyzed during the current study are available from the corresponding author upon reasonable request.

## Funding

This work was supported by grants from the 10.13039/501100004160Mazandaran University of Medical Sciences, Sari, Iran, record no: 3291, 6078.

## Declaration of competing interest

I hereby declare that I have no pecuniary or other personal interest, direct or indirect, in any matter that raises or may raise any conflict of interest.
